# Military and Civilian Sector Practice Patterns for Short-Term Travelers’ Diarrhea Self-Treatment in Adults

**DOI:** 10.4269/ajtmh.21-1037

**Published:** 2022-02-21

**Authors:** David R. Stagliano, Huai-Ching Kuo, Jamie A. Fraser, Indrani Mitra, Eric C. Garges, Mark S. Riddle, David R. Tribble, Patrick W. Hickey

**Affiliations:** ^1^ Department of Pediatrics, Walter Reed National Military Medical Center, Bethesda, Maryland;; ^2^ Infectious Disease Clinical Research Program, Department of Preventive Medicine and Biostatistics, Uniformed Services University of the Health Sciences, Bethesda, Maryland;; ^3^ Henry M. Jackson Foundation for the Advancement of Military Medicine, Bethesda, Maryland;; ^4^ Department of Preventive Medicine and Biostatistics, Uniformed Services University of the Health Sciences, Bethesda, Maryland;; ^5^ Department of Internal Medicine, University of Nevada, Reno School of Medicine, Reno, Nevada;; ^6^ Department of Pediatrics, Uniformed Services University of the Health Sciences, Bethesda, Maryland

## Abstract

The Deployment and Travel Medicine Knowledge, Attitude, Practice and Outcomes Study investigates the various clinician and traveler contributions to medical outcomes within the U.S. Military Health System. Travelers’ diarrhea is among the most common travel-related illnesses, making travelers’ diarrhea self-treatment (TDST) important for traveler health. A cohort of 80,214 adult travelers receiving malaria chemoprophylaxis for less than 6 weeks of travel were identified within the U.S. Department of Defense Military Health System Data Repository. Associated prescriptions for TDST medications within 2 weeks of chemoprophylaxis prescriptions were identified. Prescription patterns were compared by service member versus beneficiary status and site of care, military facility versus civilian facility. At military facilities, medical provider demographics were analyzed by clinical specialty and categorized as travel medicine specialists versus nonspecialists. Overall, there was low prescribing of TDST, particularly among civilian providers and military nonspecialists, despite guidelines recommending self-treatment of moderate to severe travelers’ diarrhea. This practice gap was largest among service member travelers, but also existed for beneficiaries. Compared with nonspecialists, military travel medicine specialists were more likely to prescribe a combination of an antibiotic and antimotility agent to beneficiaries, more likely to provide *any* form of TDST to service members, and more likely to prescribe azithromycin than quinolones when using antibiotics. Our study suggests that enhancing provider knowledge and use of travelers’ diarrhea treatment recommendations combined with improved access to formal travel medicine services may be important to increase the quality of care.

## INTRODUCTION

The Deployment and Travel Medicine Knowledge, Attitude, Practice and Outcomes Study (KAPOS) investigates medical provider and traveler contributions to medical outcomes within the U.S. Military Health System. ^1^ Travelers’ diarrhea is among the most common of travel-related illnesses, and travelers’ diarrhea self-treatment (TDST) is a recommended topic in pretravel healthcare visits. ^2^^–^ ^4^ The U.S. military considers travelers’ diarrhea to be one of the most important infectious disease threats to force health protection, and as such, it is a major focus of research and development activities. ^5^ Guidelines for the use of TDST exist, with many of the underlying studies conducted in a military setting. ^6^^–^ ^9^ Studies predating these guidelines show inconsistent awareness among military clinicians of travelers’ diarrhea management principles. ^10^^,^ ^11^ The aim of this study was to describe prescription patterns for travelers’ diarrhea in the Military Health System and identify variation in practice by practice setting (military versus civilian facilities), military duty status (service members versus other beneficiaries), and provider specialty (travel medicine specialists versus nonspecialists).

## METHODS

The Military Health System provides comprehensive medical care to approximately 9.5 million service members and beneficiaries worldwide through a network of 51 military hospitals, 424 military clinics, and a national and international network of participating civilian TRICARE medical providers. ^12^ This includes 1.4 million active duty service members, 0.3 million members on reserve status, more than 2 million family members of active and reserve personnel, and 5.4 million retired military and their families. The Military Health System utilizes an electronic medical record that sends administrative and clinical data into an integrated administrative record system called the Military Health System Data Repository. Containing data from both military and civilian medical facilities caring for patients of the Military Health System worldwide, the Military Health System Data Repository offers a rich repository for epidemiologic analyses. ^12^ The Military Health System Data Repository and its component data files, the Pharmacy Data Transaction Service, serve as the foundational dataset for this study.

Because long-term travelers may be more likely to have ready access to local healthcare systems, this study focuses on short-term travelers who may be more reliant on self-treatment of conditions such as travelers’ diarrhea. The study population for this analysis is a subset of travelers aged 18 years and older, previously identified as having been prescribed malaria chemoprophylaxis between fiscal years 2012 and 2016 (October 2011–September 2016). ^1^ Individuals receiving malaria chemoprophylaxis prescriptions lasting less than 6 weeks of travel duration were selected for inclusion. Unique prescriptions, not including refills, were used to define a pretravel health encounter and associated short-term trip. Prescriptions for medications associated with travelers’ diarrhea, defined as azithromycin, any quinolone antibiotic, rifaximin, loperamide, and/or diphenoxylate-atropine, dispensed within 2 weeks of the index malaria chemoprophylaxis prescription were recorded. Presence or absence of these prescriptions was the primary outcome of interest. Medical expenses and performance reporting system (MEPRS) codes were used to determine the medical specialty of the prescribing clinic from military facilities. Analysis of antimotility prescriptions was restricted to those from military facilities, and not performed for civilian sites because of over-the-counter status.

Prescription patterns were compared by site of care and military duty status. For military facilities, clinician demographics were further analyzed by clinical specialty. Civilian clinical specialty was not available for analysis. Military facilities are defined as prescriptions filled at either a military facility or a TRICARE-associated Veterans Affairs hospital; civilian facilities include prescriptions from nonmilitary medical facilities, which include mail order or retail pharmacies. Duty status categories are defined as “service members,” including active duty, National Guard and Reserve personnel; compared with “beneficiaries” consisting of retirees and dependents of service members or retirees. Within the Military Health System, infectious disease (adult and pediatric), preventive medicine, and allergy-immunology clinics typically train and use providers with formal travel medicine education. Because these clinics provide dedicated travel medicine services as part of routine operations, we defined them for the analytic purposes of this study as “travel medicine specialists.”

Data analyses were performed on SAS version 9.4 software (SAS Institute Inc., Cary, NC). Descriptive statistics were generated for all categorical variables using Pearson’s χ^2^ test. An alpha level of 0.05 was used to determine statistical significance. Odds ratios (OR), with 95% CI, were calculated to determine the magnitude of difference in antibiotic prescription patterns between facilities, duty status categories, and by travel medicine specialists versus nonspecialists. Trend graphs were generated to highlight the pattern of antibiotic usage between facilities and clinician type for the entire study duration. Deployment and Travel Medicine Knowledge, Attitude, Practice and Outcomes Study (IDCRP-097) is approved by the Uniformed Services University of the Health Sciences Institutional Review Board.

## RESULTS

During the 5-year study period, across both military and civilian medical facilities, 80,214 unique adult study subjects (Supplemental Figure 1) had 100,270 pretravel health encounters corresponding to short-term trips that met the inclusion criteria. Subjects received a median and interquartile range of 1 (1–2) prescriptions for TDST medications. As shown in Table 1, 70,838 (88%) subjects received prescriptions originating at military facilities and 9,376 (12%) subjects received prescriptions from civilian facilities. About 50,770 (63%) subjects were service members and 29,444 (37%) were beneficiaries. A majority of subjects (78%) were adults aged 18–49 years and male (71%). Across the entire cohort, subjects at 76,043 encounters (76%) were not prescribed any form of TDST for travelers’ diarrhea (data not shown).

**Table 1:** The demographic characteristics of study subjects

	Military facilities (N = 70,838)		Civilian facilities (N = 9,376)	
	Service members, n = 49,818 (70%)	Beneficiaries, n = 21,020 (30%)	Service members, n = 952 (10%)	Beneficiaries, n = 8,424 (90%)
Age (years), n (%)				
18–49	48,231 (96.8)	10,705 (50.9)	879 (92.3)	2,713 (32.2)
50–64	1,463 (2.9)	6,244 (29.7)	72 (7.6)	2,480 (29.4)
> 64	124 (< 1)	4,071 (19.4)	1 (< 1)	3,231 (38.4)
Gender,* n (%)				
Male	42,366 (85.0)	10,399 (49.5)	718 (75.4)	3,568 (42.4)
Female	7,452 (15.0)	10,620 (50.5)	234 (24.6)	4,856 (57.6)

*One subject is missing gender information.

The proportion of pretravel encounters, stratified by facility type, associated with prescription of antibiotics as part of TDST are presented in Table 2. Antibiotic TDST was infrequently (5.9–7.7%), but equally prescribed to service members during travel medicine encounters at military facilities and civilian facilities (OR 1.3, 0.99–1.6). However, antibiotic TDST was more likely prescribed to beneficiaries at military facilities compared with civilian facilities (OR 2.8, CI 2.6–3.0).

**Table 2:** Antibiotic use stratified by duty status and facility type

	Presence or absence of antibiotic* prescriptions	Military facilities, n = 89,021 (88.8%)	Civilian facilities, n = 11,249 (11.2%)	Military vs. civilian facilities, OR (95% Cl)
Service member encounters, n = 63,996 (63.8%)	With antibiotic* prescriptions, n = 4,701 (7%)	4,639 (7.7)	62 (5.9)	1.3 (0.99–1.6)
	Without antibiotic* prescriptions, n = 59,295 (93%)	58,300 (92.6)	995 (94.1)	Ref.
Beneficiary encounters, n = 36,274 (36.2%)	With antibiotic* prescriptions, n = 6,848 (19%)	5,883 (22.6)	965 (9.5)	2.8 (2.6–3.0)
	Without antibiotic* prescriptions, n = 29,426 (81%)	20,199 (77.4)	9,227 (90.5)	Ref.

*Antibiotic prescriptions included azithromycin, any quinolone, and rifaximin.

Because military facility prescriptions were also coded by clinic specialty type for each encounter, a comparison of the proportion of prescriptions for TDST in various military provider specialty clinics was possible. About 67,972 pretravel encounters at a military facility included TDST prescriptions with MEPRS codes to allow for stratification of care within travel medicine specialist or nonspecialist clinics (Supplemental Figure 1). About 21,049 encounters had associated prescriptions without a MEPRS code so were excluded from this subanalysis. The frequency and proportion of encounters associated with and without TDST for each duty status category based on clinical specialty are presented in Table 3. A majority (86.5%) of service member and beneficiary encounters were completed in nonspecialty clinics, although TDST was more likely to be omitted in these clinics.

**Table 3:** Treatment type stratified by duty status and clinic type at military facilities

Treatment type		Travel medicine specialty clinic, n = 9,192 (13.5%)	Non-specialty Clinic, n = 58,780 (86.5%)	Travel medicine vs. non-specialty clinic, OR (95% CI)
Service member encounters, n = 46,984 (69.1%)	No self treatment	854 (26.2)	37,518 (85.8)	0.06 (0.05-0.06)
	Antibiotics* alone	78 (2.4)	726 (1.7)	1.5 (1.1-1.8)
	Antibiotics* + antimotility†	893 (27.4)	1,655 (3.8)	9.6 (8.7-10.5)
	Antimotility† alone	1,439 (44.1)	3,821 (8.7)	8.2 (7.6-8.9)
Beneficiary encounters, n = 20,988 (30.9%)	No self treatment	1,065 (18.0)	9,839 (65.3)	0.12 (0.11-0.13)
	Antibiotics* alone	182 (3.0)	580 (3.9)	0.8 (0.7-0.9)
	Antibiotics* + antimotility†	2,679 (45.2)	1,764 (11.7)	6.2 (5.8-6.7)
	Antimotility† alone	2,002 (33.8)	2,877 (19.1)	2.2 (2.0-2.3)

MEPRS = medical expenses and performance reporting system.

*Antibiotic prescriptions included azithromycin, any quinolone, and rifaximin.

†Antimotility prescriptions included loperamide and diphenoxylate-atropine.

At military facilities, travel medicine specialists were less likely to omit TDST compared with nonspecialists for service members, 26.2% versus 85.8% (OR 0.06, CI 0.05–0.06). Although this disparity may be accounted for, in part, by differences in itinerary and in particular access to deployed medical support making TDST unnecessary, this disparity also persisted for beneficiaries who typically do not have access to this support. Travel medicine specialists did not prescribe self-treatment during 18.0% of beneficiary encounters compared with 65.3% of beneficiary encounters with nonspecialists (OR 0.12, CI 0.11–0.13). Antimotility agents alone, or in combination with antibiotics, for TDST were prescribed to beneficiaries by travel medicine specialists more often than by nonspecialists (OR 2.2, CI 2.0–2.3 and OR 6.2, CI 5.8–6.7, respectively). Travel medicine specialists were more likely to prescribe *any* form of TDST than nonspecialists to service members (Table 3). Overall, military travel medicine specialists prescribed antibiotics as part of TDST more frequently during pretravel encounters than providers in the civilian sector (OR 7.1, CI 6.6–7.7) (Supplemental Table 1).

Prescription events for TDST antibiotics are presented in Table 4, stratified by facility type. Because multiple antibiotic prescriptions may be provided for TDST during a given trip encounter, the number of prescriptions in Table 4 exceed the number of encounters displayed in Table 2. The most commonly prescribed antibiotic for TDST was azithromycin, followed by quinolones, and rarely rifaximin, which only accounted for 1% of the total antibiotic prescriptions.

**Table 4:** Antibiotic prescription patterns by facility type

	Military facilities	Civilian facilities	Total
Antibiotic prescriptions, n (%)	11,144 (89.7)	1,279 (10.3)	12,413
Azithromycin	7,090 (63.6)	958 (74.9)	8,048 (64.8)
Quinolone	3,963 (35.6)	288 (22.5)	6,275 (30.6)
Rifaximin	91 (< 1)	33 (2.6)	124 (1.0)

**Figure 1.**
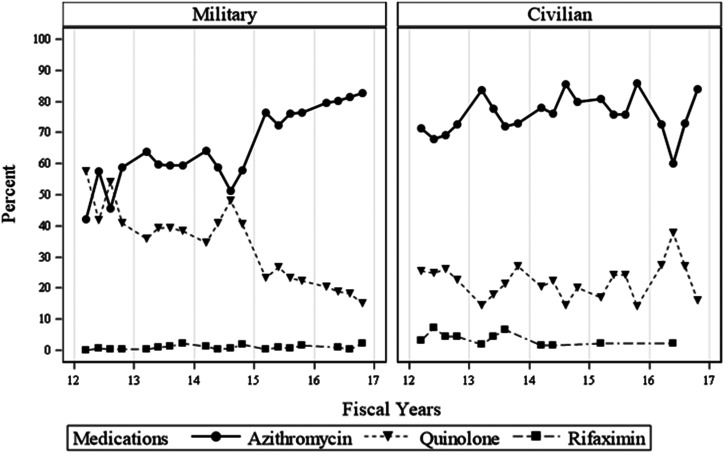
Quarterly trends of the proportion of antibiotics prescribed at civilian vs. military facilities.

**Figure 2.**
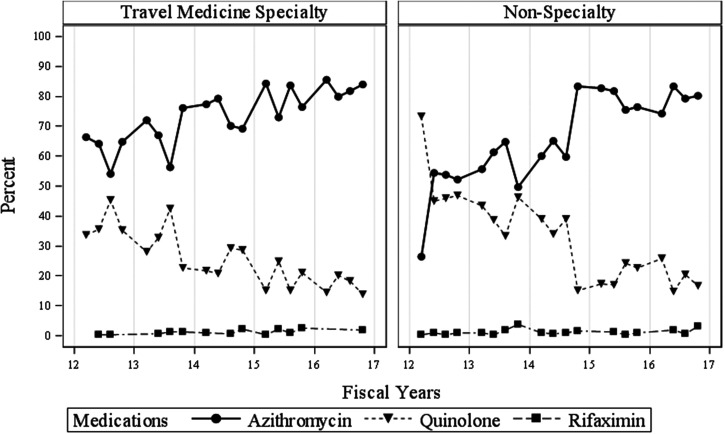
Quarterly trends of the proportion of antibiotics prescribed at military facilities between travel medicine specialists compared with nonspecialists.

Figure 1 displays the quarterly trends of the proportion of antibiotics prescribed at civilian versus military facilities. Across all time points, civilian prescriptions are represented by overall greater use of azithromycin as compared with other antibiotics with a slow increasing trend that persisted over time. In contrast, military clinicians prescribed quinolones in a roughly equivalent rate to azithromycin through the first study year, and not until the first quarter of fiscal year 2015 (October 2014–December 2014) does azithromycin become clearly preferred. Figure 2 demonstrates that within military facilities, travel medicine specialists made this shift more than a year before nonspecialists.

Among the TDST prescriptions from military facilities, 26,020 (80%) had MEPRS codes to allow for stratification by specialty type. A total of 10,867 (42%) prescriptions were from encounters by travel medicine specialists and 15,153 (58%) were from nonspecialists. About 8,745 antibiotic and 17,275 antimotility prescriptions were prescribed in travel medicine specialist or nonspecialist clinics as part of TDST. Table 5 describes the antibiotic and antimotility prescription patterns used for TDST stratified by military facility clinic specialty type. Similar to the overall prescription patterns, travel medicine specialists were more likely to provide azithromycin (OR 3.4, CI 3.2–3.5) and less likely to provide quinolones (OR 0.7, CI 0.6–0.8) than nonspecialists. With the low frequency of prescriptions for rifaximin for TDST, no statistical difference was observed between the practices of travel medicine and nonspecialists (OR 0.7, CI 0.4–1.1). Antimotility agents were also more likely to be prescribed as part of TDST in travel medicine-specialty clinics than in nonspecialty clinics (OR 3.3, CI 3.4–3.4). There was no statistical difference in the choice of antimotility agent prescribed among the specialty clinic types.

**Table 5:** Antibiotics and antimotility prescription patterns by clinic type at military facilities

	Travel medicine specialty clinic	Non-specialty clinic	Travel medicine vs. non-specialty clinic, OR (95% Cl)
Antibiotic prescriptions, n (%)	3,855 (44.1%)	4,890 (55.9%)	3.4 (3.2–3.5)
Azithromycin	2,783 (72.2)	3,130 (64.0)	1.5 (1.3–1.6)
Quinolone	1,044 (27.1)	1,711 (35.0)	0.7 (0.6–0.8)
Rifaximin	28 (< 1)	49 (1.0)	0.7 (0.4–1.1)
Antimotility prescriptions, n (%)	7,012 (40.6%)	10,263 (59.4%)	3.3 (3.2–3.4)
Loperamide	6,854 (97.7)	10,057 (98.0)	0.9 (0.7–1.1)
Diphenoxylate-atropine	158 (2.3)	206 (2.0)	1.1 (0.9–1.4)

## DISCUSSION

A predominance of young adult male service members prescribed travel-related medications is consistent with active duty demographics and mission-related travel. This serves as a noteworthy contrast to the more than 29,000 beneficiaries in this study, who would have travel patterns more similar to other civilian populations. Travelers’ diarrhea self-treatment antibiotic prescription rates were consistently low at both military and civilian settings, but antibiotics were omitted from care more often at civilian facilities. For service members, this is likely influenced by access to military medical services when deployed or traveling on temporary duty. In that context, withholding standby therapy and providing guidance on how to seek care could be an appropriate course of action. However, because the trend is also seen in care provided to beneficiaries, it suggests that patient or provider preference, or lack of knowledge of self-treatment recommendations could be factors. At military facilities, travel medicine specialists were more likely to prescribe TDST therapies compared with nonspecialists for both service members and beneficiaries. For service members, travel medicine specialists were more likely to prescribe antimotility agents, antibiotics, or combination therapy. For beneficiaries, travel medicine specialists were more likely than nonspecialists to prescribe either antimotility agents alone or a combination of antimotility agents and antibiotics. With some form of TDST prescribed to beneficiaries in approximately 82% of study encounters by travel medicine specialists versus 35% by nonspecialists, this represents an important distinction in the quality of care, based on prevailing evidence and guidelines from national organizations across the timespan that this study describes. ^7^^,^ ^9^^,^ ^13^^–^ ^15^

Before and during this study period, there was a well-described rise in quinolone-resistant pathogens that cause travelers’ diarrhea, and indeed azithromycin gradually shifted from geographically preferred agent, to a consensus recommendation. ^9^^,^ ^13^^,^ ^16^^,^ ^17^ Clinicians at civilian facilities strongly favored azithromycin as the preferred antibiotic for TDST throughout the study period. At military facilities, travel medicine specialists also demonstrated this preference, increasing their proportional use over time. Nonspecialists, however, transitioned to azithromycin use later, with a more gradual shift. This may represent a lag in the awareness of published recommendations and the evolving antibiotic susceptibility patterns for relevant travelers’ diarrhea organisms. Alternatively, this could also reflect provider preference to specifically withhold self-treatment with consideration of other guidelines recommending against general use of antibiotics for travelers’ diarrhea, concern for increasing antimicrobial resistance, concern of patient misuse of prescribed antibiotics, or acknowledgment of differing opinions among subject matter experts in travelers’ health. ^18^^–^ ^20^ In contrast to a study at Global TravEpiNet sites, which reports a decline in antibiotic prescriptions over time, our study demonstrates a relatively stable rate of antibiotic prescriptions during pretravel encounters, although the proportion of azithromycin similarly increased over time (data not shown). ^21^ Both Global TravEpiNet and KAPOS show that rifaximin is rarely used, accounting for 1% of antibiotics. Diphenoxylate-atropine was rarely prescribed, and loperamide was the antimotility agent of choice by all clinicians, though with a higher likelihood of use by travel medicine specialists versus nonspecialists (OR 3.3, CI 3.2–3.4) at military facilities.

The variations in practice described in this study are consistent with provider prescribing patterns described by Ghandi et al. with providers at academic center–affiliated sites more likely to prescribe antibiotics for travelers’ diarrhea than providers at nonacademic sites. ^21^ A key difference between Global TravEpiNet, composed of dedicated travel clinics or other clinics engaged in travel medicine to the point that they would be eligible to serve as one of 31 study sites, and KAPOS, which has access to all clinical sites for the care of a 9.7 million patients in the Military Health System population, is the insight and generalizability provided into nontravel medicine specialist, typically primary care, practice patterns. In that sense, the KAPOS analysis of both civilian site and military facility nonspecialists may offer important insights into the experience of the average American traveler. Indeed, a true understanding of the proportion of American travelers receiving pretravel care from a specialty clinic versus their primary care clinician, is not well understood. This distinction is important as other studies have shown that specialization and/or practice volume is associated with proficiency and quality. ^22^^,^ ^23^ A prior KAPOS analysis of malaria chemoprophylaxis prescribing patterns suggest that travel medicine services in the Military Health System are diffuse and often low volume on a by-provider basis. ^1^ Since nonspecialty clinics provide a preponderance of the travel-related medical care for service members and beneficiaries, the variations in practice described here reflect the need for improving the utilization of decision support tools and educational programs, along with supportive consultation from travel medicine specialists. Indeed, clinicians at military facilities have access not only to open source materials such as the CDC Yellow Book and travelers’ health website, but also, since 2008, to Shoreland’s Travax website, yet the uptake and usage rates of these tools is unknown (James Fike, personal communication). Kogelman et al. have shown that holding a Certificate of Knowledge in Clinical Tropical Medicine and Travelers’ Health from the American Society of Tropical Medicine & Hygiene, or the Certificate of Travelers’ Health in addition to the volume of travelers seen, positively influences the provision of appropriate travel advice and management recommendations. ^22^ The Department of Defense and the Military Health System have a number of formal tropical medicine and travel medicine related courses, ranging from less than 1 week courses up to a 1-year Master’s Degree program in tropical medicine. ^24^ Future studies should seek to correlate these various educational programs with quality of care by Military Health System clinicians and outcomes among patients.

The Military Health System entails a large and diverse cohort of patients, with universal access to healthcare, and a high likelihood of foreign travel, making it ideal for studying travel medicine practice and travel health outcomes. This study does have limitations. The study population was identified based on an associated malaria chemoprophylaxis prescription, and although all of these destinations are at high risk for travelers’ diarrhea, the practice patterns we observed might not fully reflect the patterns that would have been observed if we had been able to identify and include travelers to regions that were not also malaria endemic. The data reviewed was administrative claims data, not the primary electronic medical record; thus we were unable to determine the purpose and destination of travel, so we could not correlate antibiotic choices with geographic-related susceptibilities to distinguish between provider preference and adherence with temporally relevant recommendations. We also could not ascertain whether medical assets would be available to service members or beneficiaries at the destination of travel. We inferred travel duration based on the associated malaria chemoprophylaxis so it is possible that misclassification of travel duration could occur, affecting the indication for standby TDST. Although large, Military Health System patient demographics and travel context may not be generalizable, for this reason the authors would recommend focusing on beneficiary cases when drawing inference to other clinical contexts. Data from civilian site prescriptions did not include clinician medical specialty information and some MEPRS codes were missing among the military facility dataset, so we are unable to determine the relative prevalence of travel medicine specialists and nonspecialists providing care to these patients. Additionally, because antimotility agents recommended at civilian sites are over the counter and do not result in claims data, we cannot draw conclusions about antimotility medication utilization in this setting. Across all settings, patient contraindications, preference, or refusal of certain treatments may have had some effect on the results observed. Finally, Military Health System clinicians were categorized as travel medicine specialists or nonspecialists based on the specialty identification of their clinic, thus their individual familiarity with travel-specific guidelines, certification, or extent of training in travel medicine, and their overall frequency of providing travel medicine care could not be directly assessed. Understanding military and civilian provider prescribing patterns are essential to identifying opportunities for education, improving compliance with established guidelines and Force Health Protection policies, and elevating the quality of care for service members and Department of Defense beneficiaries.

## CONCLUSION

This study shows low overall prescribing of TDST, particularly among civilian providers and military nonspecialists, despite long standing CDC and other guidelines that standby self-treatment of moderate to severe travelers’ diarrhea is recommended. Variations in prescribing practices to service members may be accounted for, in part, by Force Health Protection policy, access to deployed medical support, or other differences in patient population itineraries. A practice gap also existed for both Department of Defense service members and beneficiaries cared for at military facilities. Compared with nonspecialists, travel medicine specialists at military facilities were more likely than nonspecialists to prescribe either antimotility agents alone or a combination of antimotility agents and antibiotics to beneficiaries. Travel medicine specialists were more likely than nonspecialists to prescribe *any* TDST regimen to service members. This suggests a quality of care disparity that may require enhancing provider knowledge of travelers’ diarrhea treatment recommendations, processes that improve access to travel medicine decision support tools, and consultative services.

## Supplemental Material


Supplemental materials
