# Supplementary material for: Military and Civilian Sector Practice Patterns for Short-Term Travelers’ Diarrhea Self-Treatment in Adults
Source: Am J Trop Med Hyg. 2022 Feb 21;106(4):1156–62. doi: 10.4269/ajtmh.21-1037 (PMC8991346; doi:10.4269/ajtmh.21-1037)

SUPPLEMENTARY TABLE 1. A comparison of antibiotic prescriptions by military travel medicine specialists and civilian providers

	**Military Travel Medicine Specialty Clinic n =9,192**	**Civilian Facilities n=11,249**	**Military Travel Medicine Specialty Clinic vs Civilian Facility OR (95% CI)**
**Encounters with Antibiotic[a] Prescriptions**	3,832 (41.7)	1,027 (9.1)	7.1 (6.6-7.7)
**Encounters without Antibiotic[a] Prescriptions**	5,360 (58.3)	10,222 (90.9)	Ref.

[a]Antibiotic prescriptions included azithromycin, any quinolone and rifaximin

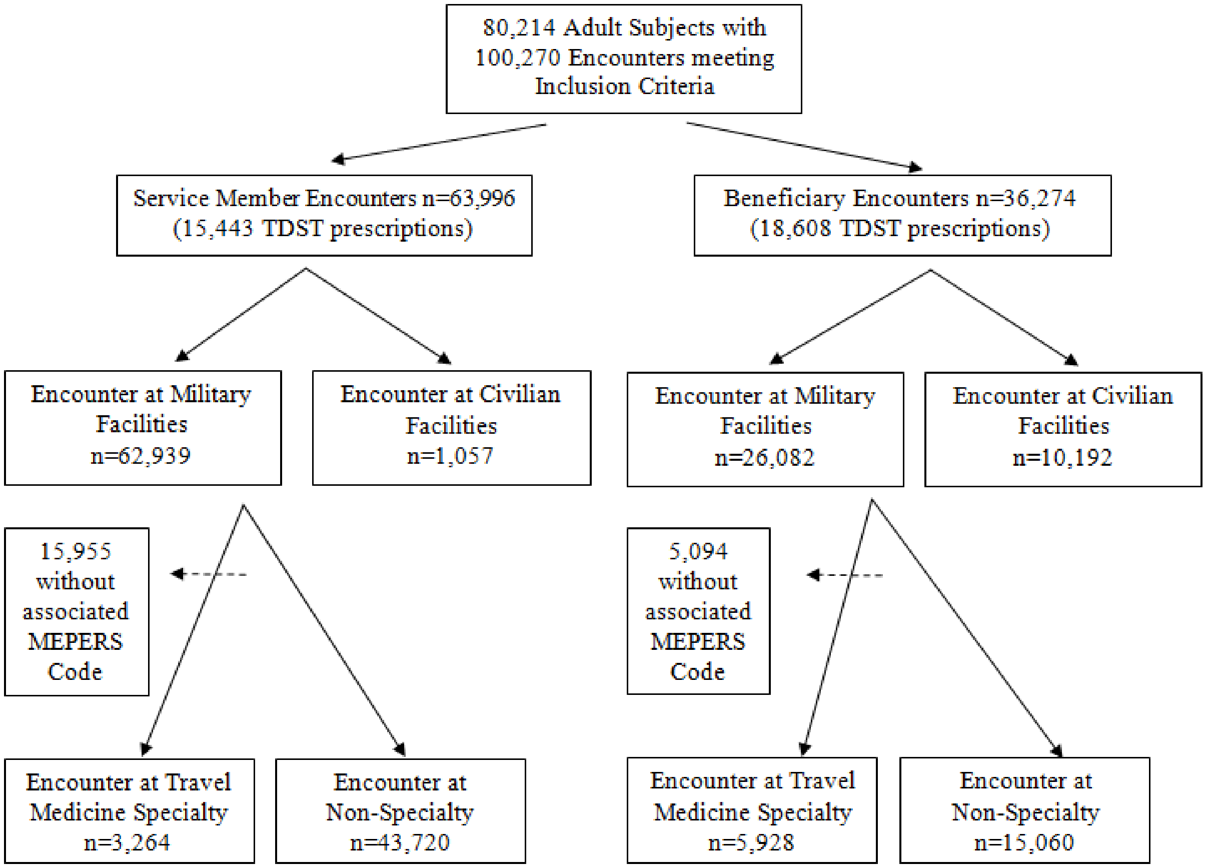

Supplement: Supplementary file 1 [file tpmd211037.SD1.pdf]
